# Point-of-Care Tests for HIV, Related Coinfections, and Blood-Borne Infections

**DOI:** 10.1155/2014/625082

**Published:** 2014-05-06

**Authors:** Nitika Pant Pai, Rosanna W. Peeling, Bryce D. Smith, David Dowdy

**Affiliations:** ^1^Division of Clinical Epidemiology, Department of Medicine, McGill University Health Center, McGill University, V Building, Royal Victoria Hospital, 687 Pine Avenue West, Montreal, QC, Canada H3A 1A1; ^2^Clinical Research Department, London School of Hygiene & Tropical Medicine, Keppel Street, London WC1E 7HT, UK; ^3^Division of Viral Hepatitis, Centers for Disease Control and Prevention, Atlanta, GA 30333, USA; ^4^Department of Epidemiology, Johns Hopkins Bloomberg School of Public Health, 615 N. Wolfe Street, E6531, Baltimore, MD 21205, USA

In the past 2 decades, rapid and point-of-care tests (POCTs) for HIV have facilitated a rapid increase in the uptake of HIV testing in sub-Saharan Africa, Asia, and the United States. POCTs for HIV are now commercially available to either detect early infection or ascertain degree of immunosuppression and monitor disease progression, thereby improving patient management. Some rapid HIV tests have evolved into POCTs, and some POCTs have even progressed into over-the-counter self- or home-based tests. In addition to the availability of unique rapid tests for hepatitis C, hepatitis B, and syphilis, multiplexed POCTs can now detect these infections using a single point-of-care device. Integration of POCT with other technologies has further expanded our ability to test and treat HIV coinfections and reduce morbidity and mortality due to HIV coinfections. For example, many POCTs for hepatitis C, hepatitis B, and syphilis are now as accurate compared to laboratory-based first-line tests, offering hope of expanded access to the millions that desire timely screening in outreach settings. In addition, novel tools like CD4 POC assays that have shown promising accuracy now offer hope of expedited triage and staging for antiretroviral treatment initiation. Timely testing and treatment will further reduce loss to followup of patients.

A quick evolution in rapid and POCT technology has catalyzed global research and policy. Implementation research with rapid and POC tests has strived to integrate some of these developments. The focus is shifting to novel testing strategies and programs that have been pilot-tested in different parts of the world, thereby offering hope of expanded access, improved patient engagement, and efficient service delivery in the times to come.

As tools to optimize screening and circumvent losses to followup, POCTs stand to play a key role in detecting, diagnosing, staging, linkage to care, monitoring treatment effectiveness, and infection control at the population level.

This special issue showcases global research with POCTs for HIV and related coinfections such as hepatitis C, hepatitis B, and syphilis. It describes examples of the implementation of research programs that have employed POCTs and targeted different populations and objectives through innovative strategies achieving impressive results.

In a study from Latin America, J. Galindo-Quintero et al. report a helpful example of how a novel POC-based testing strategy could improve HIV testing and counselling. The authors elegantly demonstrated success with the introduction of an HIV testing and counseling program in low-income neighborhoods of Cali, Colombia. In this study, an innovative peer-to-peer approach using community leaders was more effective in reaching HIV-infected individuals than facility-based campaigns with trained health care personnel. Their results stress the importance of creative implementation strategies to maximize the benefit of POC tests.

Four studies evaluated POC tests in programs and strategies in North American settings: two in the United States and two in Canada. In the southern United States, A. Zinski et al. documented a POC screening program for HIV in a 5-year cohort of test seekers. Interestingly, the study found that testing at outreach settings was associated with self-reported attendance to a primary care visit in the 12 months following an HIV diagnosis. At a time when skeptics argue against the utility and impact and cost effectiveness of POC screening programs, this study highlights the importance of expanding access to POCT in outreach and other patient-centered settings.

In Colorado, A. Jewett et al. explored the facilitators and barriers to the implementation of a POC testing program for hepatitis C virus (HCV) in an STD clinic in Denver. Interestingly, the authors demonstrated that, although integration of the POC test into the clinic standard operations gave the staff the opportunity to educate their clients about HCV, the apparent effectiveness of counselling sessions was poor. This study highlights a learning opportunity to improve the integration of counseling sessions for hepatitis C and STDs that can save time and money for patients, providers, and health systems.

In a study conducted in Edmonton, Canada, J. Bergman et al. demonstrated the feasibility of a combined POCT program for syphilis and HIV in outreach settings amidst a syphilis outbreak.

In Montreal, Canada, N. P. Pai et al. demonstrated the feasibility of evaluating an innovative unsupervised HIV self-testing strategy in a low-risk population of students of a large Canadian university. The results demonstrated that students not only were capable of conducting HIV self-tests without errors but also considered self-testing convenient and affordable. These results suggest that innovative self-testing strategies tailored to preferences of populations who traditionally avoid facility-based HIV testing may be feasible and ready for larger uptake. This is the first self-testing study from Canada.

Two reviews (one narrative, the other systematic) highlight the technological and methodological landscape of HIV and syphilis POCTs. The review by M. K. H. G. Setty and I. K. Hewlett presents a narrative overview of available POCTs for HIV diagnosis, including disease staging and monitoring. The authors discuss new technologies that may shape the next generation of POC tests. They also suggest opportunities and challenges regarding the implementation of these tests in resource-limited contexts.

The last paper by Jafari et al. touches upon an area where much work remains to be done. The field of POCT is rapidly moving towards implementation research, but documentation of outcomes beyond accuracy measures (i.e., traditional sensitivity and specificity) has been very limited, with these outcomes often left to speculation the authors conducted a systematic review of syphilis POCT studies and documented inconsistencies in reporting these outcomes. They also reported a framework that could potentially standardize reporting these commonly misreported and misclassified measures (i.e., feasibility, preference, acceptability, and impact).

By compiling these papers in this special issue, we hope to enrich our readers' knowledge on recent developments in the field of rapid and POC tests. By highlighting successes and potential pitfalls and challenges, we also aim to improve the implementation and scale-up of POCT solutions worldwide.

*Nitika Pant Pai* *Rosanna W. Peeling* *Bryce D. Smith* *David Dowdy*

